# Modulation of TLR4, hBD-2, and hBD-3 expression and hepatic tissue response by systemic probiotics in experimental apical periodontitis in rats

**DOI:** 10.1590/1678-7765-2025-0859

**Published:** 2026-05-22

**Authors:** Leopoldo COSME-SILVA, Renan DAL-FABBRO, Gabrielle Cabral Melville de Souza TENORIO, José Alex da SILVA, Edilson ERVOLINO, Letícia Cabrera CAPALBO, João Eduardo GOMES-FILHO

**Affiliations:** 1 Universidade Federal de Alagoas Faculdade de Odontologia Maceió AL Brasil Universidade Federal de Alagoas (UFAL), Faculdade de Odontologia, Maceió, AL, Brasil.; 2 University of Michigan School of Dentistry, Restorative Sciences and Endodontics Department of Cariology Ann Arbor Michigan USA University of Michigan School of Dentistry, Restorative Sciences and Endodontics, Department of Cariology, Ann Arbor, Michigan, USA.; 3 Universidade Estadual Paulista Faculdade de Odontologia de Araçatuba Departamento de Dentística, Endodontia Araçatuba SP Brasil Universidade Estadual Paulista (UNESP), Faculdade de Odontologia de Araçatuba, Departamento de Dentística, Endodontia, Araçatuba, SP, Brasil.; 4 University of Detroit Mercy School of Dentistry Division of Clinical Essentials and Simulation Detroit MI USA University of Detroit Mercy, School of Dentistry, Division of Clinical Essentials and Simulation, Detroit, MI, USA.

**Keywords:** Apical periodontitis, Probiotics, Toll-like receptor, Beta-defensins, Liver

## Abstract

Apical periodontitis is characterized by a robust inflammatory response and activation of innate immunity. Probiotics have been proposed as modulators of this response, but their local and systemic effects remain unclear.

**Objective:**

To evaluate whether systemic supplementation with *L. rhamnosus* LR-04 and *L. acidophilus* LA-14 modulates the expression of TLR4, hBD-2, and hBD-3 in a rat model of experimental apical periodontitis (AP), and to assess potential histopathological alterations in the liver.

**Methodology:**

Twenty-four male Wistar rats were randomly allocated into three groups (n=8): control (AP + water), AP + *L. rhamnosus* LR-04, and AP + *L. acidophilus* LA-14. AP was induced by exposing the pulp chambers of the first mandibular molars for 30 days. From the day of induction, probiotics (109 CFU/day via oral gavage) or water (control) were administered daily. After 30 days, the rats were euthanized. Mandibles were processed for immunohistochemical analysis of hBD-2, hBD-3, and TLR4 expression, which was assessed semi-quantitatively. Liver samples were collected, fixed, and stained with hematoxylin-eosin for histopathological evaluation of lobular inflammation, necrosis, portal inflammation, and fibrosis. Data were analyzed using the nonparametric Kruskal–Wallis test at a 5% significance level.

**Results:**

No hepatic histopathological alterations were observed in any group. Probiotic supplementation resulted in significantly greater hBD-2 immunoreactivity in periapical tissues compared to the control group (P<0.05). hBD-3 expression was significantly higher in the *L. acidophilus* LA-14 group than in both the control and *L. rhamnosus* LR-04 groups (P<0.05). TLR4 expression in periapical lesions was increased in both probiotic groups relative to control (P<0.05).

**Conclusion:**

Systemic supplementation with *L. rhamnosus* LR-04 and *L. acidophilus* LA-14 enhanced the local immune response in apical periodontitis, as evidenced by upregulation of TLR4, hBD-2, and hBD-3, without inducing detectable hepatic histopathological alterations.

## Introduction

Apical periodontitis (AP) is an infectious-inflammatory disease that develops when microorganisms invade the root canal system, eliciting a host immune response that culminates in destruction of the periapical tissues.^1,2^ This process is orchestrated by dynamic interactions between endodontic pathogens and the innate immune system, including activation of pattern-recognition receptors such as Toll-like receptor 4 (TLR4) and induction of downstream antimicrobial effectors, including β-defensins^3,4^TLR4 is a key sensor of lipopolysaccharide (LPS), a major component of Gram-negative bacterial cell walls frequently associated with endodontic infections. Its engagement initiates signaling cascades that amplify local inflammatory responses.^5^ In parallel, human β-defensin-2 (hBD-2) and human β-defensin-3 (hBD-3) are antimicrobial peptides produced by epithelial and immune cells that contribute to microbial killing while also shaping the inflammatory milieu via immunomodulatory functions.^6^ Together, TLR4-driven signaling and defensin-mediated antimicrobial activity represent central components of the innate immune network that influence AP lesion development and progression.

In recent years, probiotics have emerged as potential adjuncts for controlling inflammation in a range of chronic diseases. Probiotics are defined as live microorganisms that, when administered in adequate amounts, confer a health benefit on the host. Their use in dentistry has gained attention due to their potential effects on oral microbial ecology and host immune regulation.^7^ Among the most frequently studied strains, *Lactobacillus rhamnosus* LR-04 and *Lactobacillus acidophilus* LA-14 have shown immunomodulatory activity *in vivo*, including downregulation of inflammatory mediators and modulation of innate immune responses in experimental models.^3^ Importantly, recent animal studies suggest that systemic supplementation with these strains can attenuate inflammation and reduce alveolar bone resorption associated with AP lesions.^8^ Proposed mechanisms include probiotic–host interactions that influence TLR signaling, cytokine production, and antimicrobial peptide expression, potentially promoting microbial control while limiting excessive tissue-destructive inflammation at the periapex.^8^ These findings support the concept that manipulating the gut and/or oral microbiota may shape the immune environment of periapical tissues.

Beyond causing localized tissue damage, AP may also exert systemic impacts. The liver is crucial in immunometabolic regulation and responds significantly to ongoing inflammatory stimuli. Increasing evidence indicates that persistent oral infections, including AP, can contribute to systemic inflammatory burden and may be associated with histopathological and molecular alterations in hepatic tissue.^9,10^ Such observations are consistent with the broader “oral–gut–liver-immune” axis, in which inflammatory mediators and microbial products influence distant organs.^11^ Conversely, probiotics may confer extra-oral benefits. Experimental studies have reported improved liver outcomes following probiotic administration, including attenuation of nuclear factor-κB (NF-κB)-mediated inflammatory signaling and protection against fibrotic remodeling.^5^

Based on these observations, we hypothesized that systemic probiotic supplementation modulates innate immune signaling and antimicrobial peptide responses in periapical tissues during experimental AP, while also influencing AP-associated hepatic alterations. Therefore, this study evaluated whether systemic administration of *L. rhamnosus* LR-04 or *L. acidophilus* LA-14 regulates the expression of TLR4, hBD-2, and hBD-3 in rats with induced AP. Additionally, we assessed liver histopathology to investigate potential extraoral tissue responses associated with AP and probiotic supplementation.

## Methodology

### Animals

**Figure 1:**
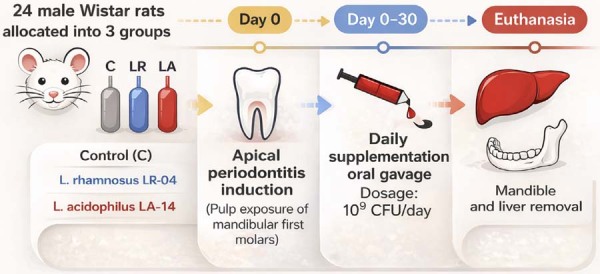
Schematic representation of the experimental design. Twenty-four male Wistar rats were allocated into three groups (n=8): Control (AP + water), AP + *Lactobacillus rhamnosus* (LR-04), and AP + *Lactobacillus acidophilus* (LA-14). Apical periodontitis was induced on Day 0 by pulp exposure of the first molars. Animals received daily oral gavage of probiotics (109 CFU/day) or water for 30 days. After euthanasia, mandibles and livers were collected for analysis.

This study was conducted in accordance with the Guide for the Care and Use of Laboratory Animals guidelines of the U.S. National Research Council. Twenty-four male Wistar rats (*Rattus norvegicus albinus*), weighing 200–250 g, were included (Figure 1). The animals were maintained in a temperature-controlled environment (22±1 ºC, 70% humidity) under a 12-h light-dark cycle, with free access to food and water.^8,12^ The experiment was approved by the Institutional Ethics Committee for Animal Use of the São Paulo State University, São Paulo, Brazil (protocol 5162017). The sample size calculation was based on previous studies.^8,13^ Assuming an alpha error of 0.05 and 95% power to detect a significant difference, seven animals per group were required. To account for a 10% loss rate, the sample size was adjusted to eight animals per group.

### Induction of Apical Periodontitis

AP was induced by pulp exposure of the first molars. Rats were anesthetized via intramuscular injection of ketamine (87 mg/kg) and xylazine (13 mg/kg). Under a surgical microscope, the coronal pulp of the first mandibular molars was accessed with a 0.5-mm round bur, and the pulp tissue was deliberately exposed to the oral cavity.^14,15^ No attempt was made to seal the access cavity, thereby enabling the oral microbiota to infect the pulp chamber and root canal. This condition was maintained for 30 days for AP to develop (Figure 1).

### Probiotic therapy

The animals were randomly assigned to three experimental groups (n=8): Control (AP + water), AP + *L. rhamnosus* LR-04, and AP + *L. acidophilus* LA-14. From the first day of AP induction, the probiotic groups received daily oral gavage of *L. rhamnosus* LR-04 (DSM 16605, Instituto Bioquímico, Campo Grande, Brazil) or *L. acidophilus* LA-14 (Aché Laboratórios Farmacêuticos, Guarulhos, Brazil). According to the manufacturers, a dose of 10^9^ colony-forming units (CFU) was contained in 0.07 g of *L. rhamnosus* LR-04 powder and in one capsule of *L. acidophilus* LA-14. Immediately before administration, the contents of one capsule (lyophilized powder) were freshly diluted in 5 mL of sterile water and administered by oral gavage. The probiotic suspensions were prepared immediately prior to use and were not stored after dilution, thereby minimizing potential loss of bacterial viability. The suspension was administered once daily by oral gavage throughout the 30-day experimental period. The control group received 5 mL of water by oral gavage on the same schedule.^16^

### Histopathological analysis of the liver

After 30 days of AP development (with or without probiotics), all rats were euthanized by anesthetic overdose. The livers were harvested and fixed in 10% neutral-buffered formalin, then processed using routine histological methods. Paraffin-embedded liver samples were sectioned at a 5-μm thickness and stained with hematoxylin and eosin (H&E). For each liver, three non-consecutive sections were obtained from the left lateral lobe, following standard procedures for experimental hepatic histopathology. In each section, five non-overlapping microscope fields were evaluated. A board-certified pathologist, blinded to group assignments, examined the sections under light microscopy for signs of lobular inflammation, hepatocellular necrosis, portal tract inflammation, and fibrosis (including pericellular, portal, or bridging fibrosis).^10^ Each liver was qualitatively assessed for the presence or absence of these histopathological changes.

### Immunohistochemical analysis

After euthanasia, the mandibles (containing the first molars) were removed and fixed in 4% buffered formaldehyde for 24 hours. Specimens were decalcified in 17% EDTA (pH 8.0) for several weeks and then embedded in paraffin. Semi-serial sections (6 μm thick) were obtained from the periapical region of the distal root of the first molar.^14^ Immunohistochemical analysis was performed using an indirect immunoperoxidase technique on deparaffinized sections.^8,14^ Sections were incubated with primary antibodies against hBD-2, hBD-3, or TLR4 (each at 1:100 dilution). A negative control was prepared by omitting the primary antibody. Immunoreactivity in the periapical region was visualized as brown cytoplasmic or extracellular staining. For each animal, three semi-serial sections of the periapical region were analyzed. In each section, five representative non-overlapping microscopic fields were evaluated by a blinded examiner under light microscopy using a digital imaging system.^14^ Staining intensity and distribution were evaluated semi-quantitatively using a 4-point scoring system.^14,17^ Immunoreactivity was scored as follows: 1 = absence of immunoreactive cells or staining (0 positive cells); 2 = low immunoreactivity, defined as few positively stained cells (1–25 cells) and faint extracellular staining; 3 = moderate immunoreactivity, characterized by a moderate number of positive cells (26–125 cells) and moderate matrix staining; and 4 = high immunoreactivity, defined as a high number of positively stained cells (>125 cells) and intense matrix staining. All histological and immunohistochemical analyses were performed by a single blinded examiner using standardized scoring criteria. Prior calibration was conducted to ensure consistency of assessments throughout the analysis.

### Statistical analysis

Data were analyzed using GraphPad Prism 10. Data normality was assessed, and the immunohistochemical scores did not meet the assumptions of normality. Therefore, comparisons among groups were performed using the nonparametric Kruskal–Wallis test, with a significance level set at 5%.

## Results

### Liver analysis

**Figure 2:**
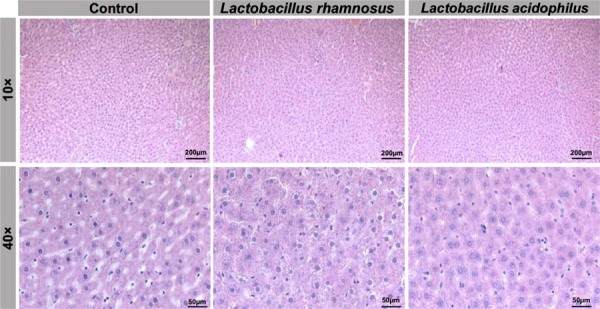
Representative histological images of hepatic tissue from the Control (AP + water), *Lactobacillus rhamnosus* (LR-04), and *Lactobacillus acidophilus* (LA-14) groups. No lobular inflammation, hepatocyte necrosis, portal tract inflammation, or fibrosis was observed in the Control, LA-14, or LR-04 groups. The liver parenchyma exhibited preserved architecture and normal morphological features across all groups. Hematoxylin and eosin (H&E) staining.

Examination of H&E-stained liver sections revealed no histopathological differences among the three groups (Figure 2). All rats, regardless of treatment, showed normal hepatic architecture, with no evidence of lobular inflammatory infiltrate, hepatocyte necrosis, portal tract inflammation, or fibrosis (Table 1). Thus, 30 days of AP did not induce detectable liver damage, and probiotic supplementation produced no observable adverse effects on hepatic tissues.

**Table 1:** Liver histological features and immunohistochemical scores for hBD-2, hBD-3, and TLR4. All groups showed no signs of lobular inflammation, hepatocyte necrosis, portal tract inflammation, or fibrosis. Immunohistochemical analyses display the distribution of scores (1-4) and corresponding medians for each marker.

	Feature	Control	*Lactobacillus acidophilus*	*Lactobacillus rhamnosus*
	Lobular inflammation	absent	absent	absent
**H&E - Liver**	Hepatocyte necrosis	absent	absent	absent
	Inflammation of portal tract	absent	absent	absent
	Fibrosis	absent	absent	absent
**IHC - AP Scores**	**IHC Marker**			
1		0/8	0/8	0/8
2		0/8	0/8	0/8
3	***hBD-2***	8/8	0/8	3/8
4		0/8	8/8	5/8
Median*		**3^a^**	**4^b^**	**4^b^**
1		0/8	0/8	0/8
2		0/8	0/8	0/8
3	***hBD-3***	8/8	4/8	8/8
4		0/8	4/8	0/8
Median*		**3^a^**	**3.5^b^**	**3^ac^**
1		0/8	0/8	0/8
2		0/8	0/8	0/8
3	***TLR4***	6/8	0/8	0/8
4		2/8	8/8	8/8
Median*		**3^a^**	**4^b^**	**4^b^**

'Different letters indicate statistically significant differences within rows (P<0.05).

### Immunohistochemical Results

**Figure 3:**
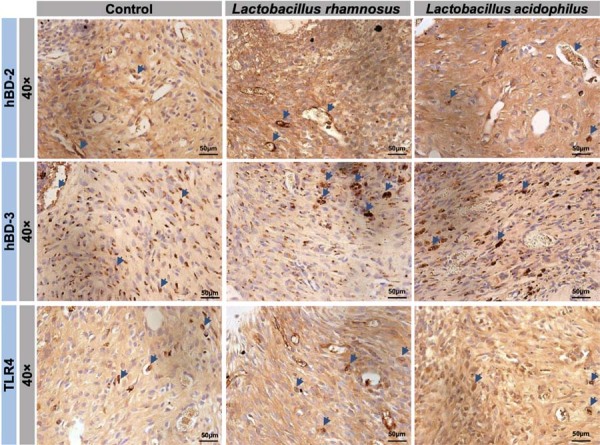
Photomicrographs illustrating the immunolabeling patterns of hBD-2, hBD-3, and TLR4 in periapical tissues from the Control (AP + water), *Lactobacillus rhamnosus* (LR-04), and *Lactobacillus acidophilus* (LA-14) groups. Arrows indicate areas of positive immunostaining. Immunoreactivity for hBD-2 and TLR4 was increased in both probiotic-treated groups compared with the Control group. Immunolabeling for hBD-3 was higher in the LA-14 group than in both the LR-04 and Control groups. Original magnification: 400×.

In the periapical tissues, immunohistochemical analysis revealed significant differences in TLR4 and β-defensin expression between the control and probiotic-treated groups (Figure 3). The control group exhibited relatively low hBD-2 immunoreactivity (median score=3), whereas both the *L. rhamnosus* LR-04 and *L. acidophilus* LA-14 groups showed high hBD-2 immunoreactivity (median score=4). The increase in hBD-2 expression in probiotic-treated rats was statistically significant compared to controls (*P*<0.05). For hBD-3, the *L. acidophilus* LA-14 group showed the highest immunostaining intensity (median score ~3.5), which was significantly higher than that observed in the control group (median=3; *P*<0.05) and the *L. rhamnosus* LR-04 group (median=3; *P*<0.05). No significant difference in hBD-3 was found between the *L. rhamnosus* LR-04 group and the control group (*P*>0.05). TLR4 followed a pattern similar to that of hBD-2: control lesions exhibited moderate TLR4 immunoreactivity (median score=3), while both probiotic-supplemented groups showed significantly higher TLR4 expression (median=4; *P*<0.05 versus control). No significant difference in TLR4 scores was observed between the two probiotic groups. A summary of the immunohistochemical scoring for all markers is presented in Table 1.

## Discussion

In this study, we demonstrated that systemic probiotic supplementation modulates innate immune markers within periapical lesions without inducing detectable hepatic injury. To our knowledge, this is the first study to evaluate, within the same experimental AP model, both local periapical immune mediators (TLR4, hBD-2, and hBD-3) and histopathological outcomes in a distant organ (liver). The selection of *Lactobacillus rhamnosus* LR-04 and *Lactobacillus acidophilus* LA-14 was based on prior evidence supporting their immunomodulatory activity and protective effects in models of oral inflammatory disease.^7,8^ Lactobacilli can exhibit antimicrobial and antioxidant properties and influence host immune responses, collectively contributing to the limitation of inflammation-driven tissue injury.^18^ Consistent with these findings, both probiotic strains increased the immunoreactivity of key innate immune components in the periapical region.

Probiotic therapy has received growing attention in oral medicine due to reported benefits on microbial homeostasis and host inflammatory regulation.^8,19^ In periodontal disease models, several *Lactobacillus* strains have been associated with reduced inflammatory infiltrate and attenuation of alveolar bone loss, supporting the notion that probiotic-driven immune modulation can preserve dentoalveolar structures.^20^ In the context of AP, the findings of this study extend this framework by showing that systemic probiotics upregulate host-defense mediators in periapical lesions, suggesting a shift toward an immune environment that may improve infection containment. Although lesion size and bone loss were not directly assessed in this study, the observed increases in antimicrobial peptide expression raise the possibility that probiotics may facilitate microbial control and contribute to a more regulated inflammatory response during AP progression and potentially during post-treatment healing.

The experimental design employed reflects a well-established rat model of AP, in which pulp exposure enables natural infection by the oral microbiota and the development of chronic periapical lesions over approximately 30 days.^7,8^ This approach reproduces key aspects of endodontic pathogenesis, including sustained microbial challenge and persistent periapical inflammation. Probiotics were administered daily by oral gavage starting on the day of pulp exposure.^6,8^ This protocol ensured consistent dosing and supported the evaluation of systemic effects while minimizing the confounding influence of direct local application, which could artificially alter periapical marker expression.^8^ Collectively, these methodological choices provided a controlled framework to assess whether systemic probiotic supplementation modulates innate immune responses in an established AP model.

A notable aspect of this study was the evaluation of liver histopathology as an indicator of potential extraoral consequences of chronic AP and of probiotic safety. Chronic oral infections have been proposed to contribute to systemic inflammatory burden, and some studies suggest that inflammatory oral conditions may influence hepatic physiology by means of immune and microbial mediators.^9-11^ Conversely, probiotics have been explored as adjuncts in liver-related conditions due to their ability to modulate gut microbiota composition, strengthen epithelial barrier function, and reduce exposure to circulating endotoxins and inflammatory signaling.^21^ In this study, however, liver sections from all groups showed preserved architecture, with no evidence of lobular inflammation, hepatocellular necrosis, portal inflammation, or fibrosis. These findings suggest that 30 days of AP in otherwise healthy rats may be insufficient to produce overt hepatic histopathology and, importantly, that the probiotic regimens tested did not induce adverse hepatic changes.

The absence of detectable liver alterations should be interpreted within the experimental context. Hepatic involvement associated with chronic oral infection may depend on factors such as longer disease duration, higher inflammatory burden, repeated infectious challenges, or pre-existing metabolic vulnerability. The gut–liver–immune axis is dynamic, and histopathological findings may lag behind molecular or biochemical changes. Thus, while our findings are reassuring regarding safety and do not indicate liver injury within the evaluated time frame, further studies with longer follow-up periods and the incorporation of systemic inflammatory and hepatic biomarkers are warranted to more comprehensively define extraoral effects of AP and the potential systemic benefits of probiotics.

At the lesion level, one of the most relevant findings was the increased expression of β-defensins in probiotic-treated animals. β-defensins are crucial components of epithelial innate immunity, contributing to microbial killing while also influencing immune cell recruitment and cytokine signaling. We focused on hBD-2 and hBD-3 because they are widely implicated in oral host defense and exhibit partially distinct antimicrobial activity profiles.^22-24^ In this model, both probiotic strains increased hBD-2 immunoreactivity, whereas LA-14 produced the highest enhancement of hBD-3 compared with both the control group and LR-04. This strain-dependent pattern suggests that different probiotic organisms may engage host signaling networks by means of distinct mechanisms, leading to differential regulation of antimicrobial peptide expression. Mechanistically, probiotics can stimulate antimicrobial peptide production via pattern-recognition receptor-mediated pathways, as well as by indirect effects on immune regulation and microbial competition. Regardless of the precise upstream trigger, increased local availability of β-defensins may strengthen antimicrobial defense at the periapical interface and support more effective containment of the polymicrobial challenge associated with AP.

Probiotic supplementation also increased TLR4 immunoreactivity in periapical lesions. Because TLR4 is a principal sensor of LPS and can drive NF-κB-dependent pro-inflammatory cascades, increased TLR4 expression might initially appear counterintuitive if probiotics are expected to “reduce inflammation.” However, expression levels alone do not necessarily indicate harmful overactivation. Increased TLR4 immunoreactivity may reflect enhanced pathogen-sensing capacity or immune “priming,” enabling earlier recognition and more coordinated innate responses. In our study, elevated TLR4 levels co-occurred with increased β-defensin expression and were not associated with evidence of systemic tissue injury. These findings support the interpretation that probiotics may promote a more competent and regulated local innate response rather than simply amplifying destructive inflammation. Future studies incorporating cytokine panels, pathway activation assays, and bone/lesion outcomes would help clarify whether TLR4 upregulation in this context corresponds to beneficial immune regulation.

From a translational perspective, the ability to modulate innate immune mediators in AP is relevant because persistent microbial challenge and dysregulated inflammation are key drivers of periapical tissue breakdown and impaired healing. An immune profile characterized by robust antimicrobial effector expression combined with controlled inflammatory signaling could, in principle, promote more effective microbial clearance and lesion resolution. Systemic probiotics are a practical adjunctive strategy that may complement conventional endodontic therapy, particularly in cases in which host immune competence or systemic inflammatory load may influence healing parameters. While these implications are promising, they remain speculative given the current study design, which did not quantify lesion size, bone resorption, or post-treatment healing outcomes.

Several limitations should be acknowledged. First, immunohistochemistry was assessed using a semi-quantitative scoring system, which is suitable for comparative analyses but does not provide absolute quantification of protein expression. Second, although the 30-day period was sufficient to establish AP, it may have been too short to detect extra-oral consequences in otherwise healthy animals; systemic inflammation may occur in the absence of histologically detectable liver changes. Third, systemic endpoints (e.g., circulating cytokines, endotoxin levels, gut permeability markers, hepatic enzymes, or microbiome profiling) were not assessed, thereby limiting mechanistic interpretation of the gut–immune–liver axis.

## Conclusion

Systemic supplementation with *L. rhamnosus* LR-04 and *L. acidophilus* LA-14 increased periapical expression of TLR4 and β-defensins, supporting the notion that probiotics can modulate innate immune defenses during experimental AP. Within the study timeframe, AP did not produce detectable hepatic histopathology, and probiotic administration was not associated with adverse liver effects. Future studies should combine quantitative molecular analyses with functional outcomes (including lesion size, bone resorption, microbial profiling, and post-endodontic healing) and include systemic biomarkers to better define the mechanisms and clinical relevance of probiotic-driven immunomodulation in AP.
